# A mathematician’s guide to plasmids: an introduction to plasmid biology for modellers

**DOI:** 10.1099/mic.0.001362

**Published:** 2023-07-28

**Authors:** Ian Dewan, Hildegard Uecker

**Affiliations:** ^1^​ Research Group Stochastic Evolutionary Dynamics, Department of Theoretical Biology, Max Planck Institute for Evolutionary Biology, Plön, Germany

**Keywords:** plasmids, modelling, bacterial evolution, mobile genetic elements, horizontal gene transfer

## Abstract

Plasmids, extrachromosomal DNA molecules commonly found in bacterial and archaeal cells, play an important role in bacterial genetics and evolution. Our understanding of plasmid biology has been furthered greatly by the development of mathematical models, and there are many questions about plasmids that models would be useful in answering. In this review, we present an introductory, yet comprehensive, overview of the biology of plasmids suitable for modellers unfamiliar with plasmids who want to get up to speed and to begin working on plasmid-related models. In addition to reviewing the diversity of plasmids and the genes they carry, their key physiological functions, and interactions between plasmid and host, we also highlight selected plasmid topics that may be of particular interest to modellers and areas where there is a particular need for theoretical development. The world of plasmids holds a great variety of subjects that will interest mathematical biologists, and introducing new modellers to the subject will help to expand the existing body of plasmid theory.

## Data Summary

**Fig. 1.**
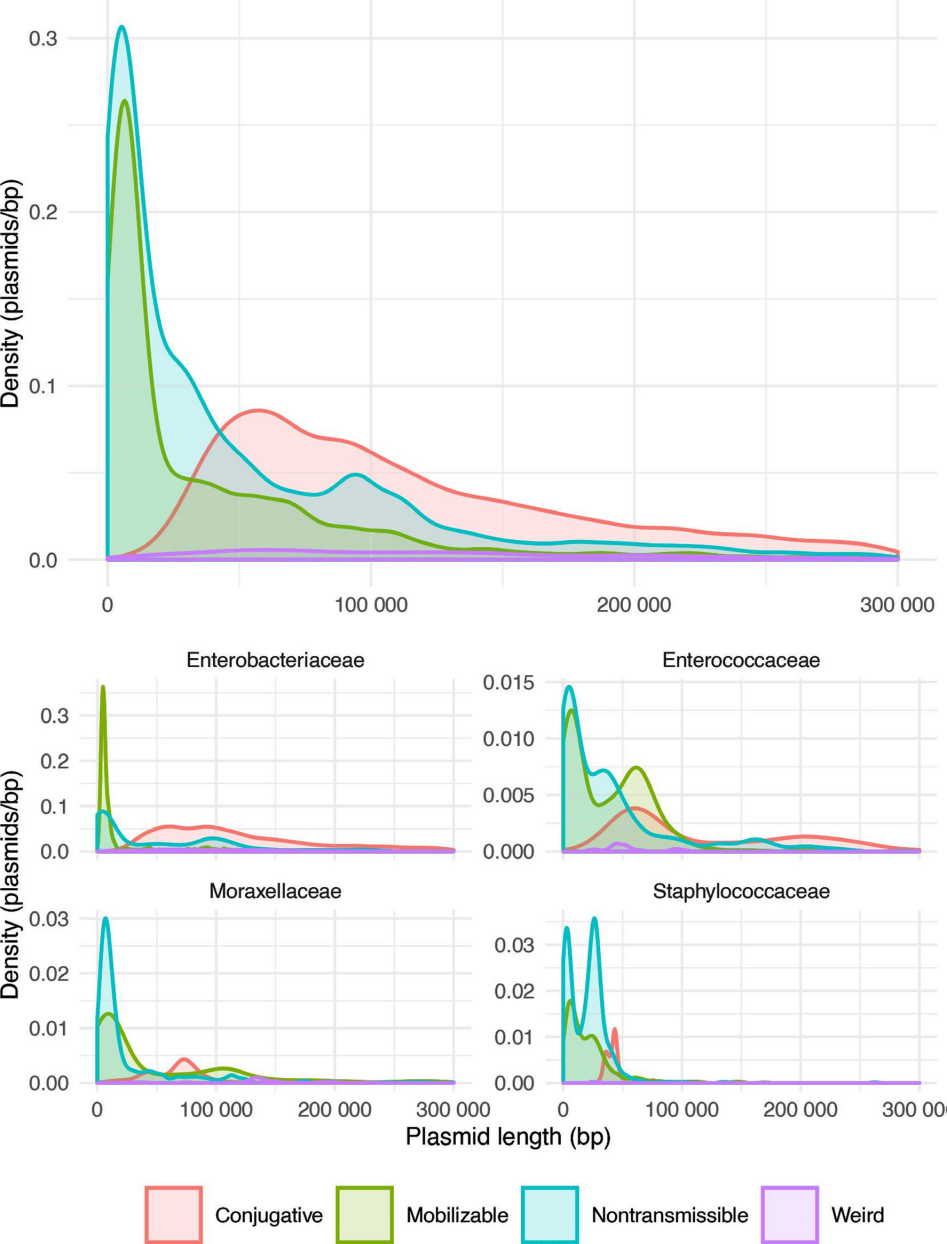
Distribution of plasmid lengths among mobility classes of plasmids. Plasmid length data are taken from PLSDB (retrieved 1 March 2023 [237]) and the distribution is derived by kernel density estimation in R [238] with the ggplot2 library [239]. The top panel shows all plasmids in the database; the bottom four panels show plasmids from the four bacterial families with the most sequences in the database. The graph was cut off at 300 000 bp for reasons of scale: 1844 plasmids in the database (5.34% of the total) are longer than the limit; the longest plasmid in the database is 4 605 385 bp. The plasmid sequences in PLSDB have been annotated with MOB-typer [240] to identify putative relaxases* or conjugative genes (see the explanation of these terms in the text). Those plasmids with neither are nontransmissible, those with both are conjugative, and those with only the relaxase are mobilizable; note that this means that those mobilizable plasmids with an *oriT* but no relaxase are not recognized as mobilizable. The 'weird’ class includes those plasmids that had conjugative genes but no relaxase: these would be nontransmissible, but nonetheless have all the genes for a secretion system and mating pair formation. It is possible that these are misidentified conjugative plasmids, which have an unknown relaxase, or misidentified mobilizable or nontransmissible plasmids, which do not actually have conjugative genes.

Supporting data for Figure 1 can be found at 10.6084/m9.figshare.23744571 [1]

## Introduction

Plasmids are extrachromosomal DNA molecules common in many bacteria [2]. They replicate independently from the chromosome (and from other DNA molecules in the cell), and often exist in the cell in multiple copies. They can be transmitted vertically to daughter cells on host cell division and in some cases horizontally to other bacteria. The simplest plasmids are effectively parasites of their hosts: they colonize the host and use its cellular machinery to reproduce themselves. But they also form part of the bacterial genome, and genes located on plasmids have effects on the metabolic processes of their hosts, on the host phenotype, and therefore on host fitness. The most studied of these plasmid-borne genes are antibiotic resistance genes, which are a serious threat to the continuing effectiveness of antibiotics in clinical use [3, 4].

The term ‘plasmid’ was coined by Lederberg [5] to refer to any genetic determinant outside the chromosome, including chloroplast and mitochondrial genomes and certain viruses, but is now restricted to the simple extrachromosomal DNA molecules found in bacteria, archaea and some eukaryotes. Historically, plasmids were of interest for two primary reasons: horizontal transfer between cells and antibiotic resistance. The earliest plasmids discovered were capable of transferring themselves between bacterial cells [2, p. 9], and this novel method of horizontal gene transfer was of great interest to bacterial geneticists.

Antibiotic resistance plasmids were also discovered early on: this greatly increased interest in plasmids as a clinically important contributor to the spread of antibiotic resistance [6]. Today, a much greater variety of plasmids are known and studied by biologists. As components of the bacterial genome, plasmids play an important role in the evolution of bacterial populations; therefore, a clear understanding of all aspects of plasmid biology is necessary for a full understanding of bacterial genetics.

Plasmids are also of interest in their own right, as evolving biological entities. Mathematical modelling can make, and has already made, an important contribution to understanding the biology of plasmids and their role in the ecology and evolution of bacteria. The existing literature (reviewed in [7]) includes models focusing both on fundamental plasmid biology and on particular contributions of plasmids to bacterial populations, especially antibiotic resistance. Mathematical approaches are diverse and depend on the question at hand: we briefly review them in Box 1.

While a large number of plasmid models exist, this number is still small in the light of their biological interest. For those interested in learning about plasmids, there are a large variety of existing reviews covering plasmids and their evolution generally [6, 8, 9], the diversity of plasmids [10–12], antibiotic resistance plasmids [4, 13–15], the physiological functions of plasmids [16–21], and interactions between plasmids and between hosts and plasmids [14, 22–26]. Why therefore another review article?

The diversity and complexity of plasmids can easily be overwhelming for those without prior knowledge, which includes many theoretical biologists who originally have a background in a subject other than biology, such as mathematics, physics, or computer science. In particular, as soon as a novice sets up a model, many questions about meaningful biological assumptions appear. To lower the hurdle to start working on plasmids, we here present an overview of plasmid biology explicitly targeted at modellers new to the field.

An introduction to plasmids for modellers could certainly have various levels of biological detail and complexity: here, we want to go beyond a basic and largely conceptual level. From our own experience, we mostly see the need for a review that, while introductory, does not oversimplify the biology. While no prior knowledge about plasmids is required to read the article, some background about bacteria and microbiology is assumed. In the interest of accessibility to modellers with differing knowledge of microbiology, we provide a glossary of terms used: terms in the glossary are marked with an asterisk on first use. In the next three sections, we focus on plasmids themselves, discussing the variety of plasmids, their gene content and the important physiological functions they carry out; in the fifth section, we explicitly turn to the relationship between plasmids and their hosts, considering plasmid–host interactions, as well as interactions between plasmids. In four boxes, we highlight selected key topics related to plasmids and their hosts that are especially captivating for modellers, and provide a few references to relevant modelling work as a starting point for further reading. At the end of the article, we discuss several areas where we see particular scope (and need) for more modelling. Hopefully, the review will enable readers to begin working on models that will expand our understanding of plasmids and their hosts.

Box 1. **Modelling approaches used in plasmid models**

A variety of methods have been applied to modelling plasmids and their hosts. The oldest and most widely used are deterministic differential equation models that divide the host population into groups based on plasmid content, in the simplest case just plasmid-carrying and plasmid-free bacteria. These models track the population sizes of the groups, coupling the population dynamics with the dynamics of the plasmid. This technique has been applied to exploring the effects of conjugation on plasmid dynamics [112, 220] and to the ‘plasmid paradox‘ [221–224] (discussed further in Boxes 4 and 5, respectively), as well as many other questions [69, 121, 122, 203]. Some models use difference equations in a similar fashion [182, 225]. Stochastic simulations of such models, usually with stochastic rates paralleling the transition rates of the deterministic models, are also common [225, 226]. Other models have used analytical stochastic approaches [198]; branching process models are particularly useful when modelling the invasion of a plasmid or the early spread of a novel plasmid variant [205, 227, 228]. While most plasmid models include population dynamics, some models, in the tradition of population genetics, fix the population size. Based on extensions of the classical Moran model [229], these models only consider changes in the relative frequencies of cell types: these can be deterministic [68, 230] or stochastic [230, 231]. The final common approach is the use of individual-based simulations [102, 206, 232, 233], which allow a realistic incorporation of many biological processes at the cost of little analytical tractability. Although most models are of a host population, there are some models of plasmids within a single cell, both deterministic [234, 235] and stochastic [199]. Of course, many studies combine multiple modelling methods: particularly common are the combination of an analytically tractable model of some sort with a simulation [225, 228, 233].

The biology, the specific question and the goal of the model determine which processes and features are taken into account. Models of a host population usually incorporate a few common biological processes. The most basic of these is the host population dynamics, which includes a growth model (often either exponential or Monod growth), competition between cells (often via a Lotka–Volterra model or explicit inclusion of a common resource), washout of cells (for chemostat models) and fitness costs or benefits to carrying particular plasmids. Horizontal transfer is frequently included: see Box 4 for a discussion of modelling horizontal transfer. Finally, loss of the plasmid during segregation is also a common model component: this can be modelled explicitly as a component of a stochastic model, but in ODE models it is usually modelled as a flux of cells from a plasmid-carrying to a plasmid-free compartment at a constant *per capita* rate. Other model features include physiological changes in the cells or age structure in the population [102, 112], migration [68], explicit modelling of the segregation of plasmids on host cell division [182, 228] and plasmid replication during the cell cycle [236], and many others.

The parameters associated with these models usually need to be assigned values, although for some models it may be possible to obtain analytical results (e.g. [223]). Many studies incorporate experimental and modelling work in the same study [98, 220, 232], and then the parameters can be estimated from the accompanying experiments. In the absence of codesigned experiments, parameter values or plausible parameter ranges usually need to be obtained from the literature, either directly or by additional parameter estimation from published data. For models with one or few parameters, it may be possible to explicitly explore the plausible parameter space. Otherwise, depending on the goals and scope of the study, a parameter sensitivity analysis might be required. Parameter estimates are usually obtained from *in vitro* experimental studies. Parameter estimation is often non-trivial (see also Box 4 for the estimation of transfer rates). The parameterization of *in vivo* models is notoriously difficult. An example of thoughtful and sophisticated parameter estimation for an *in vivo* model is the method developed by Tepekule *et al*. [226], who make use of various kinds of data and observations from multiple different sources, including microbiome time series data, to parameterize their model.

## Natural history of plasmids

Plasmids are extremely widespread in the wild: bacteria and archaea from almost all taxonomic groups have been found to contain plasmids [8]. They also exist in eukaryotes, particularly fungi (see e.g. [27, 28]), and even in mitochondria within eukaryotic cells [29]; but we shall focus our attention on bacterial (and archaeal) plasmids. Precise quantification of the frequency of plasmids in different taxa is complicated by variable search effort: plasmids from bacteria of clinical importance or from model species are overrepresented among sequenced plasmids, particularly those from the Gammaproteobacteria, where antibiotic resistance plasmids have been the subject of extensive study [30].

**Fig. 2.**
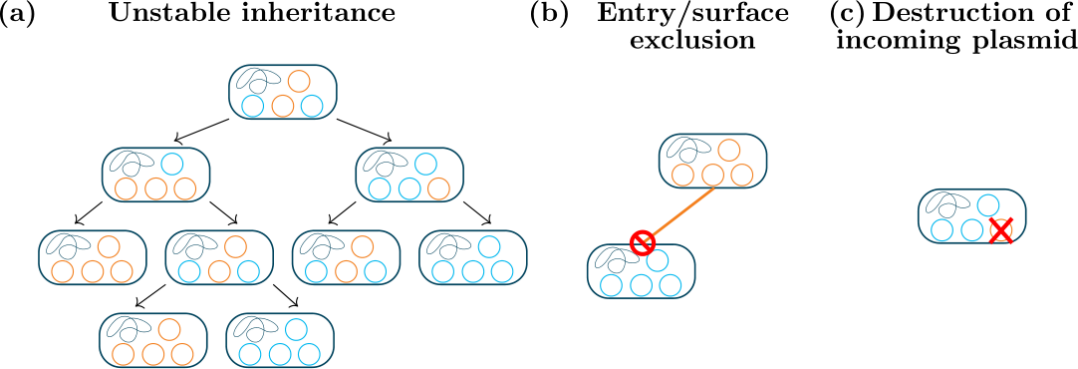
Mechanisms limiting coinfection of bacteria by multiple plasmids. (a) Unstable inheritance. Because the two plasmid types share a common copy number, random segregation at host cell division eventually ensures that they are separated into distinct hosts carrying only one plasmid type. (b) Surface and entry exclusion. The plasmids encode proteins which prevent the host cell from being a recipient in conjugation. (c) Destruction of the incoming plasmid. Novel plasmids are degraded by plasmid-encoded immune systems, such as CRISPR/Cas* or restriction enzymes* once in the host cell.

Plasmids themselves may be naturally classified on the basis of biologically relevant properties. Perhaps the most important of these is the capacity for horizontal transmission* between bacteria by conjugation (the details of conjugation are discussed below). Conjugative plasmids carry all of the genes necessary for conjugation, and are therefore self-transmissible; mobilizable plasmids do not carry the full transfer machinery, but have a sufficient subset to be able to undergo conjugation in the presence of a conjugative plasmid which supplies the rest; the remaining plasmids are nontransmissible. It has been estimated that about half of all plasmids are nontransmissible, with the remaining half approximately equally divided between conjugative and mobilizable [31, 32]; however, recent results suggest that a large fraction of plasmids that are traditionally classified as nontransmissible might in fact be mobilizable, and mobilizable plasmids might make up the majority of all plasmids [33]. The capacity for conjugation is connected to two other biologically relevant properties: plasmid size and the number of copies of the plasmid maintained in a host cell. In general, conjugative plasmids are large and have a low copy number (typically one or a few copies per cell), while small plasmids, which tend to have a high copy number, are more often mobilizable or nontransmissible [8]: see Fig. 1. Nontransmissible plasmids exhibit a much larger range of sizes than transmissible plasmids, and there are some nontransmissible plasmids that are even larger than typical conjugative plasmids, which may be in the process of becoming accessory chromosomes* [31, 34]. Plasmids may also be classified as broad or narrow host-range plasmids, depending on whether they are capable of becoming established in a large variety of hosts, or are reliant on a particular group of bacteria [16]. Plasmids are also classified based on topology, which has particular effects on the biology of replication: the majority of plasmids are circular, but some linear plasmids have been found [35–38]. Beyond this division of plasmids based on broad properties, it is natural to develop a systematic biological classification of plasmid types. The classical approach is to divide plasmids into *incompatibility groups*. We say that two plasmids are in the same incompatibility group if they are incompatible; that is, if they cannot be stably maintained together in the same cell line [39]. This concept is perhaps a bit counterintuitive – plasmids are in the same group if they are incompatible with each other – but it produces natural kinds because the cause of incompatibility is usually interference between common regulatory systems on the incompatible plasmids [20]: this mechanism of incompatibility is shown in Fig. 2(a), and discussed further below. Because incompatible plasmids share fundamental genes, incompatibility groups may be looked on as ‘species’ of plasmids, and the production of new incompatibility groups as plasmid speciation [40, 41]. It has been suggested that in the modern world of abundant plasmid sequences, the concept of incompatibility groups could be replaced altogether in favour of directly comparing sequences of fundamental plasmid genes; this has been argued to address perceived conceptual limitations, such as the ability of single point mutations to create new incompatibility groups [42, 43]. These methods have used the sequences of the replication proteins (REP classification), the conjugative transfer proteins (MOB classification), or the entire plasmid to reconstruct phylogenetic relationships between plasmids [10, 44–47]. We shall see below that plasmids can fuse with each other and separate again, and they gain or lose segments by the movement of mobile genetic elements or by recombination: therefore the concepts of a plasmid type and the identity of a plasmid over time can be quite fluid in practice, with plasmids that belong to multiple incompatibility groups, or that significantly change their structure or gene content while remaining in the same incompatibility group. For the purposes of modelling, whether different plasmids count as being of the same type will generally simply have to be imposed by the modeller.

## Anatomy of plasmids

The understanding of plasmids must of course start with the plasmid itself, as a molecule, a sequence of nucleotides, and a collection of genes.

### Genes carried on plasmids

The genes carried on a plasmid may be broadly divided into two groups based on their function: those with plasmid-specific functions and those that primarily affect the host phenotype. The genes responsible for plasmids’ own housekeeping functions make up the plasmid backbone, while the other genes carried by a plasmid are called payload or accessory genes [16]. The housekeeping genes of the plasmid typically fall into a few classes: there are the genes responsible for the replication of the plasmid, a stability system that ensures the plasmid is stably inherited across host generations and, if the plasmid is transmissible, the genes responsible for conjugation or mobilization and overcoming host defences to establish the plasmid in the recipient cell [11]. The most important of these are the genes controlling replication. They will include the *oriV* (origin of vegetative replication, the sequence at which plasmid replication begins), as well as the genes for any proteins necessary for replication and for the components of a system to control replication of the plasmid [11]. Typically, the origin of replication and a few genes involved in replication form the minimum subsequence of the plasmid that is capable of replication in a host, called the ‘basic replicon’*, which constitutes an absolutely minimal plasmid [48]. The simplest plasmids – often called cryptic plasmids – may have no payload genes [49–51]; they therefore do nothing but hang around in the cell. But many plasmids, and for obvious reasons the plasmids of greatest interest, carry genes that contribute to the phenotype of the host cell. The most studied plasmid payload genes are antibiotic resistance genes. These are found extremely frequently, in a wide variety of hosts, and encoding resistance to a wide variety of antibiotics [3, 4, 52]; some aspects of plasmid-borne antibiotic resistance and models thereof are discussed in Box 2. Plasmids may also encode resistance to other environmental dangers, such as heavy metals or other toxins [53–55]. A second large class of payload genes provide some new metabolic process [56, 57]; this might include, for example, the ability to metabolize a new substrate for growth [58–60]. Virulence factors are also often found on plasmids [61]. Plasmids also carry genes responsible for social interactions between bacteria (discussed below). Particularly interesting examples of plasmid-borne traits include the gall-forming properties of the plant pathogen *Agrobacterium tumefaciens *, which are caused by the horizontal transfer of a portion of a plasmid to the host plant [62], and the symbiosis between nitrogen-fixing rhizobial bacteria and leguminous plants, in which both the genes responsible for nitrogen fixation and for interactions with the plant host are found on plasmids [63]. An extensive list of functions observed on plasmids may be found in [1, pp. 4–5]. Sometimes, even essential genes are found on plasmids [64, 65], although extrachromosomal replicons with the unique copy of an essential gene are sometimes categorized as secondary chromosomes* or chromids* instead of plasmids. Why particular genes are found on plasmids rather than on chromosomes is a longstanding question. Numerous hypotheses and models have been developed to explain the distribution of genes (see e.g. [63, 66–69]). Possible explanations include the advantages of mobility for local adaptation when there is patchy or temporally varying selection [63, 66]. In different conditions the same genes may be located on different replicons: it has been hypothesized that the majority (if not all) bacterial genes have spent time on plasmids and on chromosomes over evolutionary time [66].

Box 2. **Plasmids and antibiotic resistance**

Antibiotic resistance genes are frequently located on plasmids [241, 242], and the serious threat of antibiotic resistance means that understanding the contribution of plasmids to its evolution is one of the most important applications of plasmid biology. The reasons antibiotic resistance genes are so often found on plasmids may include that plasmids allow bacteria to temporarily acquire resistance genes that are not needed in nonselective environments [52], or that the presence of plasmids in multiple copies increases either the dosage of resistance genes [243, 244], or the rate of emergence of new resistance alleles [90, 245], or both. Horizontal transmission of plasmids enables antibiotic resistance to spread very quickly in bacterial communities [4, 13, 246]. The human gut is a site of substantial plasmid transfer. Resistance plasmids within the gut continue to evolve [247] and are transferred between members of the microbiota within individuals and spread across individuals, as shown by León-Sampedro *et al*. [248] for the plasmid pOXA-48 in the gut of hospital patients. During antibiotic treatment, not only the pathogen population but also the patient’s normal microbiome is exposed to antibiotic pressure, inadvertently selecting for resistance (so-called bystander selection). However, resistance plasmids are also found in human populations without strong exposure to antibiotics [249]. Resistance in commensal bacteria can be problematic for two reasons. First, some commensal species, such as *Escherichia coli *, *Klebsiella pneumoniae *, or *Staphylococcus aureus *, can turn pathogenic and cause infections of the urinary tract, the lung, or wounds, and are a major cause of nosocomial infections. Second, resistance plasmids may spread from commensal bacteria to obligate pathogens [250–253]. Despite the immense clinical relevance of plasmids, modelling studies are biased towards chromosomal resistance. Nonetheless, a body of theoretical literature on plasmid-mediated resistance has accumulated [15, 254], and plasmids are increasingly attracting the attention of modellers. For example, Svara and Rankin [207] developed an ODE model to determine under which treatment conditions – antibiotic dose and intervals between administrations – resistance on a conjugative plasmid would be favoured over resistance on the chromosome. Their model also includes an incompatible plasmid without the resistance gene, leading to six different cell types that compete with each other. Other models have, for example, addressed how patterns of drug use determine the prevalence of resistance plasmids within the microbiota at the level of the individual [226] and the level of the population [254] and examined the interaction of plasmid-borne resistance with specific modes of drug action [212]. Models furthermore often complement *in vitro* experiments to help explain and interpret the observed dynamics of plasmid-mediated antibiotic resistance (e.g. [245, 255]).

### Genetic structure

**Fig. 3.**
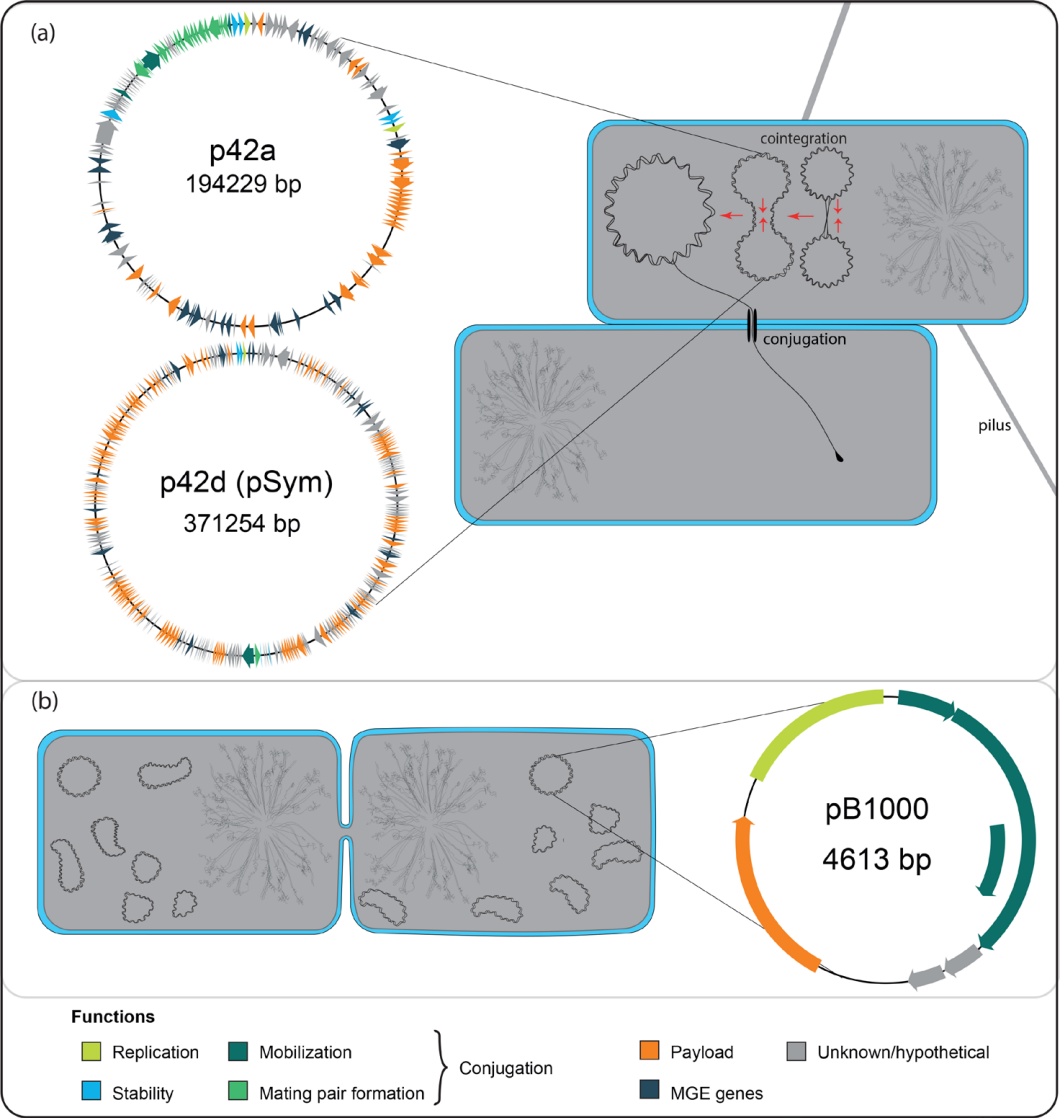
Illustration of important processes in plasmid biology with examples of three different plasmids. (a) The low-copy-number (~1 copy per cell) plasmids p42a (conjugative) and p42d (nontransmissible) of *Rhizobium etli* CFN42 (GenBank accession numbers CP000134.1 and U80928.5 [256, 257]) fuse into a cointegrate and then are transferred by conjugation from a host to a new recipient. The two cells have been pulled together by a pilus, which has then been retracted. A single strand of a plasmid copy is transferred, while the other strand remains within the cell in circular form. Afterward, a second strand will be synthesized in both cells to generate double-stranded DNA. p42d is also referred to as pSym, since it contains most of the genes responsible for rhizobial symbiosis in this strain; it is normally transferred horizontally by cointegration with p42a [191, 258]. (b) The copies of the small, high-copy-number (~14 copies per cell) resistance plasmid pB1000 (GenBank accession number GU080070.1 [180]) have been segregated between daughter cells at host division; cell division is almost complete. Inside each bacterial cell, the plasmids and chromosome are depicted. The diagrams of the plasmids show the open reading frame* of each gene on the plasmid as an arrow coloured by the function of the encoded protein. The region of pB1000 marked in the replication colour contains the plasmid origin of replication and the coding sequences for the RNAs involved in regulation of replication. The origins of replication of p42a and p42d are located inside the RepA gene; the origin of transfer of p42a is marked with a dot in the mobilization colour.

The genes on plasmids are not distributed arbitrarily, but tend to have an organized structure. This structure is often modular, with genes with related functions located together on the plasmid; there are then segments of the plasmid for replication, conjugation, different payload genes, and so on [11]; see, for example, the plasmids depicted in Fig. 3. In addition, the genes carried on plasmids are often located within other, nested mobile genetic elements*: a plasmid-borne gene may be in a gene cassette*, which is integrated into an integron*, which is carried by a transposon*, which is located on the plasmid [16]. The modular structure suggests that plasmids may frequently evolve by the gain or loss of entire modules, whether by recombination* with chromosomes or other plasmids or by the movement of mobile genetic elements. This also means that plasmids are frequently genetic mosaics, which may combine components of several mobile genetic elements together with pieces of multiple original plasmids and chunks of chromosomes [70–75]. Large plasmids may contain multiple basic replicons (e.g. F, see [76]); this means that they can replicate starting from any one of their several *oriV* sequences using the replication mechanism encoded by that basic replicon. Usually, only one will be active at a time, since otherwise there would be conflicts between the replication processes; often different replicons will be active in different hosts, extending the plasmid host range [12].

### Plasmid multimers

In addition to the formation of mosaics by accumulation of portions of plasmids and other replicons, several plasmid copies may fuse into one molecule, forming a plasmid multimer; plasmids frequently exist in hosts as a mixture of monomers and multimers of various sizes [77–80]. Multimerization occurs not only between plasmids of the same type, but also of distinct types, forming plasmid cointegrates that exhibit the properties of both their components [72, 81–84]: this is depicted in Fig. 3(a). The formation of plasmid multimers and cointegrates is driven by homologous recombination* or recombination of transposable elements located on one or the other plasmid [85–87], and sometimes by more exotic processes (reviewed in [22]); to counteract multimerization, plasmids frequently encode multimer resolution systems.

## Physiology of plasmids

The backbone genes of plasmids have functions related to the ‘life’ of the plasmid itself, and are responsible for its maintenance in the host, its propagation (vertically or horizontally) and similar housekeeping. These functions constitute the physiology of the plasmid, and their presence is what distinguishes a plasmid from a simple fragment of DNA that might be picked up by transformation*.

### Replication and plasmid copy number and their control

It is a defining attribute of plasmids that they replicate in their host cells autonomously from the chromosome. Although most plasmids rely partially on host proteins for replication, they also carry genes essential to their replication and responsible for its regulation. The regulation of replication ensures that the plasmid is present in the host population at a fixed number of copies per cell. Control of copy number is necessary to ensure the stable maintenance of the plasmid: if the copy number falls too low, there is a greater risk of producing plasmid-free segregants, while if the copy number is allowed to grow without limit, the plasmid will impose a heavy fitness cost on its hosts; Box 3 discusses the multilevel selection acting on traits such as plasmid copy number. We have already seen that there are low- and high-copy-number plasmids; but there is much copy number variation among high-copy-number plasmids, from around 10 copies to hundreds (e.g. [78, 88]). The copy number is not exactly identical from cell to cell, but is subject to some noise, which can be reduced by a partitioning system [89]. The plasmid copy number may of course be subject to evolution, even over short time scales when the plasmid carries an important host function [90–92].

Box 3. **Plasmids and multilevel evolutionary dynamics**

**Multilevel selection**

Because plasmids depend on a bacterial host to survive and propagate, they offer an excellent example of multilevel selection: the fitness of plasmids depends both on their own properties (of replication, maintenance, etc.) and indirectly on the fitness of their hosts [66]. As we have seen, plasmid-borne genes frequently affect host fitness as well as the fitness of the plasmid itself: this includes both direct effects on host phenotype and the indirect fitness effects discussed in the text. Interactions between these two levels have important implications for the evolution of plasmids and for their contribution to the evolution of their hosts. For example, there is a trade-off between rates of vertical and horizontal transmission of plasmids [15, 100, 128]: the costs to the host of conjugation mean that increased horizontal transmission reduces vertical transmission. A similar trade-off also leads to conflicting selection pressures on the plasmid copy number: a plasmid variant with a higher copy number outcompetes a variant with a lower copy number at the intracellular level, but if the copy number increases too much, the burden on the host cell may become too high [129, 259].

**Allele dynamics on multicopy plasmids**

**Fig. 4.**
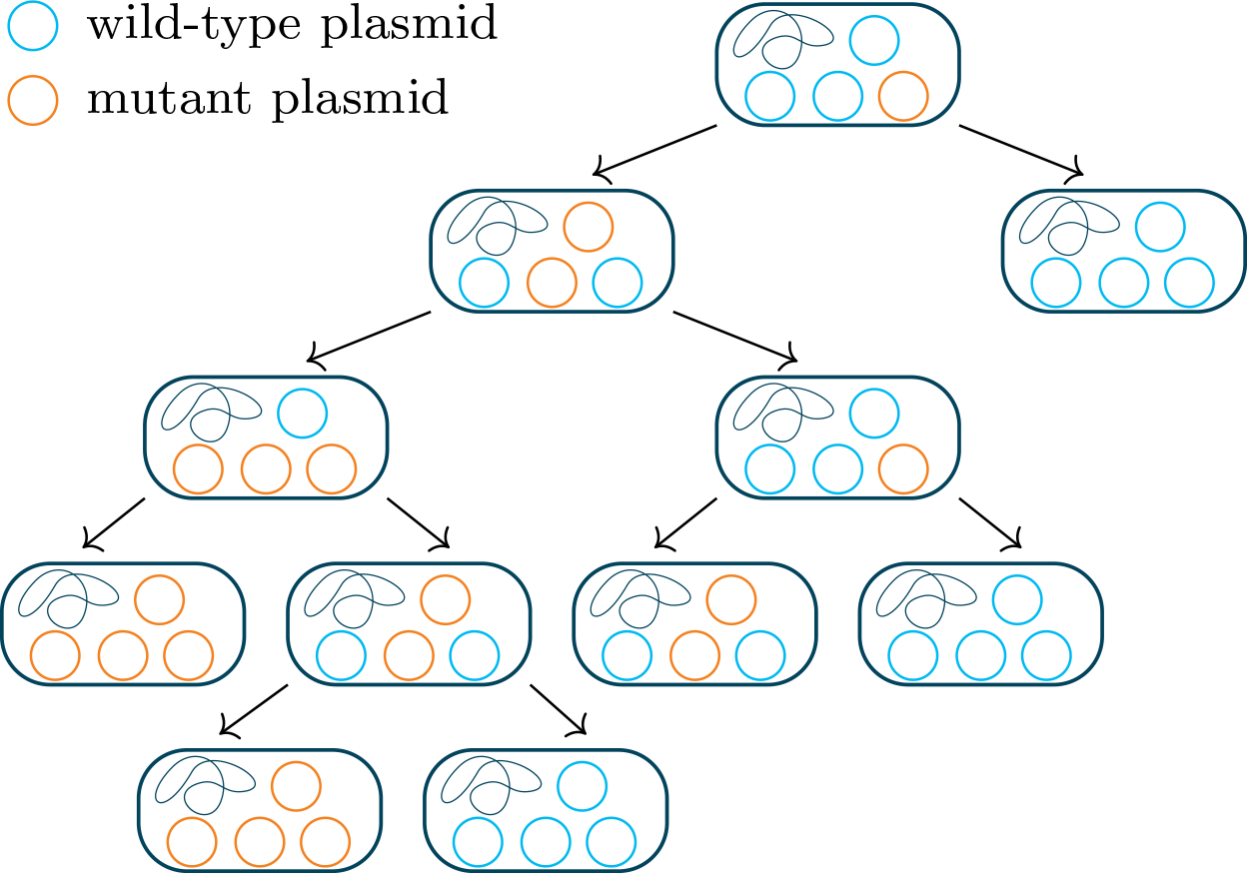
Schema of the effect of segregational drift on a novel allele located on a plasmid. A novel allele originally arises on one copy of the plasmid, and by random segregation wild-type and mutant plasmids eventually end up in different, homozygous cells. We here describe one way of modelling the process mathematically [228]. In the simplest case, cell dynamics are modelled by a birth-death process with *per capita* birth and death rates $b_{i}$ and $d_{i}$ , which may depend on the number of mutant plasmids i in a given cell. Prior to cell division, plasmids are replicated such that the cell contains twice the original number. Two possible models for plasmid replication are ‘regular replication’, where each copy is replicated exactly once, and ‘random replication’, where copies are randomly picked for replication one by one (cf. [198]). Each daughter cell then receives half of those plasmids with segregation being random with respect to mutant and wild-type variants. With *n* total plasmid copies and *x* mutant copies after plasmid replication, the probability that one daughter cell receives *j* and the other one *x−j* mutant copies is then given by (2−δj,x/2)(xj)(2n−xn−j)/(2nn) , where $\delta_{i,j}$ denotes Kronecker’s delta. Adapted from [90, 245].

An effect of multicopy plasmids on bacterial evolution is the possibility for loci on multicopy plasmids to be heterozygous (reviewed in [8]). The dynamics of alleles on multicopy plasmids depend on processes at two levels – intracellular plasmid replication and segregation and population dynamics at the cellular level. If the alleles have fitness effects, the host fitness depends on the plasmid composition within the cell, and various forms of dominance or heterozygote advantage are possible. As illustrated in Fig. 4, random segregation of plasmid copies at host cell division changes the plasmid composition from mother to daughter cells, which is termed segregational drift [260]. In the long term, this leads to the loss of heterozygosity on plasmids unless there is selection for maintaining the two plasmid types together. Segregational drift does not alter the allele frequency in the bacterial population per se, but since it leads to the generation of wild-type homozygous cells, it reduces the number of cells carrying the mutant allele, which increases the strength of genetic drift. This means novel mutations on plasmids have a higher chance of being lost than those on a monoploid chromosome, at least in the absence of gene dosage effects [98, 228, 261]. Replication of plasmids may also be a source of drift: selection of plasmids to replicate, if at least partially random, creates a ‘rich-get-richer’ effect that leads to more extreme biases in plasmid content in offspring [230]. The evolutionary dynamics on multicopy plasmids have recently received substantial attention from both experimentalists [90, 261] and theoreticians [228, 230] and from both together [98, 245, 260, 262]. Early models have studied the dynamics of incompatible multicopy plasmids, which is a closely related problem [198, 200, 201].

The replication control system ensures that the replication rate per plasmid copy per host generation is greater than one when the copy number is below the target and less than one when the copy number exceeds the target [1, p. 31], so that the copy number is increased by replication or reduced as host cell divisions dilute the plasmids faster than they replicate to reach the target. The rate of replication is regulated by negative feedback: one common mechanism is by control of the production of a plasmid-encoded Rep protein that is responsible for initiating the replication of the plasmid by binding to the *oriV* and recruiting the necessary enzymes for replication. Expression of Rep is repressed by some *trans*-acting* product encoded by the plasmid: a separate, specialized repressor*, Rep itself, or another protein cotranscribed with Rep. As the plasmid copy number increases, more of this repressor is produced, and it prevents the production of Rep protein, creating a negative feedback loop [2, 17, 18, 93–95]. This mechanism explains the origin of plasmid incompatibility due to a common basic replicon. Since the repressors are *trans*-acting, two distinct plasmids with the same basic replicon will contribute to repressing each other’s replication, and therefore will share a common copy number. In the absence of selection for both plasmid types, this means that after a few generations random segregation* at host cell division will have separated the plasmid types into distinct host cells, and thus the two plasmids cannot be stably maintained together [20]; see Fig. 2(a). When replication is permitted by the control system, the selection of the plasmid copies to be replicated seems to be at random in at least some cases [96, 97], although it is difficult to definitively confirm this experimentally, and it may not be the case for all plasmids [98]. The mechanisms of replication are reviewed in [19].

### Segregation of plasmids at host cell division

When a host cell divides, the plasmid copies present end up in one or other of the daughter cells (see Fig. 3b). In the absence of any intervention by the plasmid, this will happen essentially at random: the plasmids in one portion of the cell when it divides go to one daughter, and the plasmids elsewhere go to the other. For plasmids with a high copy number, this may be sufficient to stably vertically transmit the plasmid: with *n* copies, the probability of producing a plasmid-free daughter by random segregation is 2^1*−n*^, which may be sufficiently small to be negligible. Indeed, some high-copy-number plasmids do not seem to have any other partitioning mechanism [96]. In practice, this is quite successful in reducing plasmid loss to a very low level [99].

For low-copy-number plasmids a further active partitioning system that ensures that both daughter cells receive a copy of the plasmid is necessary. This can be done by localizing plasmids in separate regions of the cell before cell division (e.g. [100, 101]). A common class of such systems functions by encoding a protein complex and a *cis*-acting* site on the plasmid. The protein complex binds a pair of plasmids together at their respective copies of the *cis*-acting site and then physically separates the pair at cell division, possibly by binding to a point on the cell membrane or another host structure [21].

Even high-copy-number plasmids need to ensure that they are not made unstable by multimerization. The replication control systems we have discussed above control the number of copies of the basic replicon in the cell; this means that if multimers of the plasmid form, the number of physical molecules containing the plasmid falls without a derepression of replication (as the number of copies of the basic replicon remains the same). Since the number of physical molecules is what determines the probability of producing a plasmid-free daughter cell, multimerization reduces the stability of the plasmid. Moreover, larger plasmid multimers are replicated more often, since more copies of the basic replicon means more *oriV*s at which to initiate replication. A cell with multimers is thus likely to obtain more multimers, and reduce the plasmid’s stability further, in the so-called ‘dimer catastrophe’ [102]. To counteract this effect, plasmids generally encode a multimer resolution system, which converts multimers back to monomers by site-specific recombination* [21, 103, 104].

Another group of plasmid functions, variously called postsegregational killing, host killing, toxin–antitoxin systems, or plasmid addiction, are often included with stability systems, but we shall consider them separately below.

### Horizontal transfer by conjugation

Perhaps the most famous physiological function of plasmids is the ability to transfer themselves from one host to another. Transfer by conjugation requires physical contact between the donor and recipient cell, which is usually, at least in Gram-negative* bacteria, achieved using a proteinaceous structure called a pilus: see Fig. 3(a). Usually, a single strand of the plasmid is transferred into the recipient cell, and the second strand is then resynthesized in both cells, meaning that conjugation also involves duplication of the plasmid; however, in some plasmids of *Streptomyces,* the entire double-stranded plasmid is translocated from the donor to recipient [105, 106]. In some cases, there is the possibility of retrotransfer, the transfer of genetic material from the recipient back to the host, during conjugation [107]. Some plasmids can undergo horizontal transfer through more exotic mechanisms [108], sometimes involving cooperation between donor and recipient [109], but here we focus on common conjugation. In addition to the pili, which bring the donor and recipient into contact so that conjugation can occur, the typical conjugation machinery consists of four parts.

The first is the *oriT,* or origin of transfer, the sequence on the plasmid at which transfer begins. One strand of the plasmid is nicked* here by the second component, a relaxase*, which binds to the free end of the strand. The relaxase interacts with a type IV coupling protein (T4CP, the third component), and is transferred, together with the attached plasmid strand, by a type IV secretion system (T4SS, the fourth component) into the recipient cell. Once the entire strand has passed into the recipient cell, the relaxase catalyses the ligation* of the transferred strand back into a circular form [31]. This mechanism explains the distinction between conjugative and mobilizable plasmids discussed above: conjugative plasmids carry all of these components, while mobilizable plasmids encode only the *oriT* and corresponding relaxase, but not the expensive T4SS (and may or may not carry the T4CP), and therefore need a conjugative plasmid to provide the missing components. Some mobilizable plasmids only have the *oriT,* and rely on another plasmid even for a matching relaxase; these generally have a reduced rate of transfer [22, 33, 110]. The T4SS, T4CP and pili are each large protein complexes, which are costly to express, and the process of conjugation itself is energetically costly [25]. Moreover, some bacteriophages* bind to the proteins of the pilus (so-called ‘male-specific phages’), so bacterial cells expressing pili are at greater risk of infection. Therefore the conjugative phenotype is tightly regulated, to avoid imposing a large fitness cost on the host cell, and is almost always unexpressed [24, 111]. Signals that lead to expression of conjugation may include the detection of suitable recipients or specific environmental conditions [17, 111]; also, a temporary period of expression typically occurs immediately after a novel plasmid is first transferred to a recipient, before the repression becomes effective [112]. The modelling of conjugation in general, and conjugation rates in particular, is discussed in Box 4.

Box 4. **Conjugative plasmid spread**

**Fig. 5.**
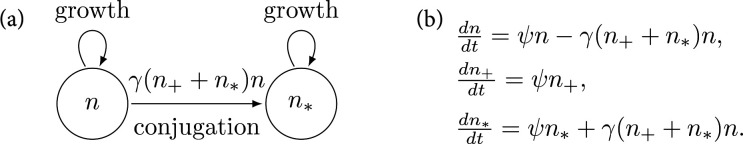
Flow diagram (a) and system of ODES (b) for the model of Levin et al. [220]. Three types of cells are distinguished: plasmid-free (with density n ), an original plasmid-carrying population (with density _$n_{+}$ _, not shown in the flow diagram) and transconjugants (with density $n_{*}$ ). Conjugation is modelled through a mass-action kinetics term with conjugation coefficient γ , and the bacterial populations grow exponentially at rate ψ .

Dating back to Stewart and Levin [221], the majority of modelling studies consider a well-mixed population (such as a liquid culture) and assume that conjugative plasmid transfer follows the principles of mass-action kinetics [263]. Transfer is thus proportional to the densities of plasmid-free and plasmid-carrying cells and a conjugation coefficient γ . The framework is similar to classical epidemiological models with infected and uninfected patients. Based on the model shown in Fig. 5, Levin *et al*. [220] showed that mass-action kinetics with a constant γ captures the plasmid dynamics well in populations with constantly dividing cells (but not during lag phase or close to stationary phase). Models based on this approach have been used to address a large range of questions on plasmid dynamics, such as the plasmid paradox described in Box 5 [223], the question of which genes are carried on plasmids [69, 264], the dynamics of cooperative genes and cheating [265], the evolution of antibiotic resistance [207, 226] (see also Box 2) and the role of conjugation in the rate of adaptation [208, 227]. A simplifying assumption in most of these models is that the transfer coefficient γ is constant in time and independent of, for example, resource availability [263]. Dependence of γ on a resource C can be modelled by a Monod function (see e.g. [266]). Spatial models of plasmid transfer are the minority. One of the first examples of a spatial model is the individual-based lattice model by Krone *et al*. [232], in which zero, one, or two cells may reside on the sites of a lattice. Plasmid-free cells receive plasmids at stochastic rates that depend on the number of donors and transconjugants* in a local neighbourhood and the amount of nutrients in a ‘nutrient neighbourhood’. The model is combined with experiments on agar surfaces, capturing the experimental observations well.

How much do plasmids conjugate? The donor and recipient species, the plasmid, environmental factors and coinfecting plasmids influence the rate of plasmid transfer [267]. There is, surprisingly, no standard method for estimating conjugation rates [268–270]. Many studies do not estimate the conjugation coefficient γ but report quantities such as the number of transconjugants per donor or per recipient. Methods to estimate the conjugation coefficient γ are mostly derived from models that describe plasmid spread by ordinary differential equations [220, 266, 269]. For example, Levin *et al*. [220] solved their ODEs (Fig. 5) to obtain the expression

$$\gamma =\frac{\psi }{N(a)-N(b)}\ln\frac{R+\rho (b)}{R+\rho (a)},$$

where N(b) and N(a) are the total population sizes at time points a and b , $\rho (t)=\frac{n_{*}(t)}{n(t)}$ and $R=\frac{n_{+}(t)}{n(t)}$ (the last of which is a constant in the model). All quantities on the right-hand side can be estimated from experimental data to obtain an estimate for γ . Recently, Kosterlitz *et al*. [268] proposed a fluctuation test similar to the fluctuation assays for mutation rate estimation, which go back to Luria and Delbrück [271]. Estimates for γ range over several orders of magnitude, ranging from ~10^-20^ ml cell^-1^ h^−1^ to 5×10^-7^ ml cell^-1^ h^−1^ [267]. It seems that conjugation rates can be higher *in vivo* than *in vitro* [272–274].

Like most – if not all – traits, the conjugation rate is itself subject to evolution [275, 276]. As the conjugation rate can be controlled by the host, the recipient cell, or the plasmid, evolution of both bacteria and plasmids can change how much transfer occurs. Selection pressures on bacteria and plasmids might thereby diverge (recently formalized in a model by Sheppard *et al*. [275]). As explained in the main text, conjugation is costly. Unless the plasmid carries genes that are beneficial to its host and outweigh the costs, selection drives the conjugation rate down. The plasmid, on the other hand, needs to strike a balance between horizontal transfer and vertical transmission [275, 276]. Higher conjugation rates are beneficial but only to the extent that the host is not harmed too much, similar to the transmission–virulence trade-off in pathogen evolution.

Transmissible plasmids often also encode genes that assist the plasmid in becoming established in newly infected cells, particularly if the cells are genetically distinct from the previous host. The region containing these genes is usually close to the *oriT* and is transferred first during conjugation, so that genes that suppress restriction enzymes* or the SOS response* in the newly infected cell can be expressed early on [11, 113]. These genes are responsible for overcoming the various components of the bacterial immune system [73, 113], which we discuss below.

Conjugative and some mobilizable plasmids prevent closely related plasmids from transferring into their hosts by conjugation (see Fig. 2b). There are two mechanisms for this: surface exclusion, which prevents mating pair formation, and entry exclusion, which is more common and prevents plasmid DNA from entering the host during conjugation (reviewed in [23, 24]).

### Postsegregational killing

Many plasmids exhibit a spiteful behaviour: they kill plasmid-free daughter cells of their hosts. This phenomenon is variously called postsegregational killing, host killing, or plasmid addiction. It was briefly mentioned above, together with stability mechanisms, because this is the classical explanation of postsegregational killing: it ensures the stable inheritance of the plasmid (see e.g. [2, 21, 114, 115]).

The postsegregational killing system involves two components (generally proteins or RNAs), one that kills (or at least suppresses growth in) the host, and a second that suppresses the first: for this reason these systems are also called toxin–antitoxin systems. Both are expressed constitutively* in the presence of the plasmid, and the antitoxin prevents the death of the host. However, the antitoxin has a much smaller half-life, so in plasmid-free daughters it degrades faster than the toxin, and the toxin kills the cell [21, 116, 117]. A slight variant is found in restriction–modification systems, in which there is no need for a difference in half-life: the antitoxin (methylase) needs to be present in a higher concentration to suppress killing than the toxin (restriction enzyme) needs to kill, and as the concentrations fall in the absence of the plasmid the antitoxin becomes ineffective first [118, 119]. A plasmid-free daughter cell is plasmid-free whether it is alive or dead; postsegregational killing can maintain the relative stability of a plasmid by ensuring that the *proportion* of hosts carrying a plasmid does not decrease, but that does not affect the *absolute number* of hosts carrying the plasmid, which continues to decrease. This is confirmed both empirically [120] and by modelling [121]. In the exponential phase of bacterial growth, therefore, postsegregational killing would have no stabilizing effect. In the stationary phase, however, where competition between hosts and plasmid-free cells for resources becomes more important, or when there is significant spatial structure [122], postsegregational killing may have a more important role in plasmid stability. Postsegregational killing may also serve other functions besides stability, including interplasmid competition [120, 123]. Toxin–antitoxin systems are also frequently found on bacterial chromosomes, where their function is somewhat obscure [117, 124–126].

## Ecology of plasmids

Thus far, we have focused on the biology of the plasmid itself, largely in isolation; however, plasmids exist in a larger ecological context that includes their hosts, the larger population of hosts (or potential hosts) and the community it is part of, and other plasmids and nonplasmid mobile genetic elements in their host or the host population. (Pilosof [127] provides a brief review of relevant concerns in modelling such complex communities.) Here, we explicitly consider the influence of interactions with all of these entities on the biology of the plasmid and host.

### Interactions with the individual host

The plasmid only exists inside the host, and depends on the host to carry out all of its physiological processes, in particular replication. As we have already seen in Box 3, this aspect of the plasmid lifestyle means that multilevel selection is of great importance to plasmids. Plasmid fitness can be divided into two components corresponding to the two ways a plasmid can propagate to new hosts: vertical, from vertical transmission to daughter cells of their current host, and horizontal, from infection of new hosts by conjugation. Vertical fitness of plasmids is directly dependent on host fitness, while horizontal fitness is more independent (although plasmid spread by horizontal transmission still requires persistence of the host). In general, there may be strong trade-offs between vertical and horizontal transmission [128, 129], for example due to the fitness costs of conjugation to the host. Since both contribute to total plasmid fitness, there will often be different possible successful life history strategies with a different emphasis on horizontal versus vertical transmission [15] (the extreme case being nontransmissible plasmids that rely entirely on vertical transmission).

The importance of the plasmid–host relationship is not unilateral. Although hosts are not dependent on plasmids (with some exceptions where essential genes are found on plasmids), plasmids form a part of the host genome, and are therefore intrinsically linked to the host phenotype and fitness. We have already discussed the wide range of payload genes found on many plasmids that provide important host phenotypes, including antibiotic resistance, virulence, metabolism of novel substrates and rhizobial symbiosis. Payload genes on plasmids may be particularly important for phenotypes that are involved in local adaptation [63, 67]. They often have context-dependent fitness effects, providing fitness benefits in some environments and conditions and causing fitness costs in others [130, 131]. In addition to carrying important genes, plasmids also frequently influence the regulation and expression of chromosomal genes, which similarly affects the host phenotype (reviewed in [132]). In some cases this can take the form of interference between plasmid and host gene regulation, producing a reduction in host fitness [133, 134], particularly for plasmids that are new to the host and not well adapted. However, changes in the expression of chromosomal genes can also be adaptive, affecting traits such as motility, biofilm formation, antibiotic resistance, virulence, or aspects of the bacterial metabolism [26, 132]. Nevertheless, in the absence of genes with important host fitness effects, plasmids are often costly to the bacteria that carry them, and this cost increases with copy number [90, 135]; the fitness costs of plasmids lead to the so-called ‘plasmid paradox’ discussed in Box 5. In addition to interference between gene regulation or the physiological processes of the plasmid and chromosome [14, 25, 133, 134, 136–139], the causes of this fitness cost include bacterial stress responses to foreign DNA [14, 25], the cost of plasmid replication and gene expression [14, 25, 140, 141] and the possibility of disruption of host genes by the plasmid recombining with the chromosome [25]. These costs can be reduced or eliminated by compensatory evolution of the plasmid [137, 142–144], the host chromosome [145–150], or both [138, 151–154], and evolution experiments have shown that this occurs regularly, especially if selection for the plasmid ensures that it is maintained before compensation is complete [138, 142–146, 148, 149, 151–153, 155, 156]; there is also some observational evidence of compensatory evolution in plasmids in the wild [157, 158]. The coevolved plasmid and chromosome produced by compensatory evolution are specifically adapted to each other, and to existence as components of a single genome [26] (although some compensatory changes on the plasmid are general enough to also reduce the fitness cost in other hosts [152]), even to the extent that coevolved cells cured of the plasmid have lower fitness than wild-type cells [145, 159, 160]. Gama *et al*. [14] argue that this coevolution – or rather its loss – leads to the overestimation of the fitness costs of plasmids in laboratory conditions compared to those that are common in nature: the reported *in vitro* fitness cost of a plasmid seems to increase with time since plasmid isolation, suggesting that the fitness costs seen in experiments may be at least partially due to maladaptive plasmid evolution under laboratory conditions, leading to the loss of evolved cost mitigation (see also [135]). As already pointed out above, the fitness cost of the plasmid may also be strongly dependent on the environmental context [131], further reducing the generalizability of experimental measurements.

Box 5. **The plasmid paradox**

One of the great questions of plasmid biology is the ‘plasmid paradox’: why are there plasmids at all? A simple cryptic plasmid, which does nothing but replicate itself inside the host, imposes a fitness cost. Therefore, over the long term, bacteria carrying plasmids should be outcompeted by plasmid-free bacteria and go extinct, taking their plasmids with them [159, 160]. In some cases, plasmids may transfer horizontally fast enough to be maintained despite fitness costs, analogously to how parasites survive [160], although reliable estimates for transfer rates in nature are needed to determine whether conjugation rates are in fact high enough. It is difficult to see, however, how horizontal transfer could maintain mobilizable plasmids, and of course it is impossible that horizontal transmission explains the maintenance of nontransmissible plasmids.

Plasmids could also be maintained by carrying advantageous genes that would make the net fitness effect of the plasmid positive: but these genes could move by recombination from the plasmid to another replicon such as the chromosome, allowing the bacterium to lose the plasmid and have its cake and eat it too. Bergstrom *et al*. [223] analysed an ODE model of plasmid persistence, starting from the assumption that the rate of horizontal transfer is not high enough to maintain the plasmids as parasites: this assumption is formally given by the relation

,$$\psi \alpha +\tau §gt;\gamma N^{+}$$

where ψ is the *per capita* growth rate of plasmid-free bacteria, α is the proportional fitness cost of carrying the plasmid, *τ* is the *per capita* rate of segregative loss, *γ* is the horizontal transfer rate and $N^{+}$ is the maximum total bacterial population size obtainable. They show that this implies the eventual extinction of plasmid-carrying cells, even if there is a beneficial gene on the plasmid (or fluctuating selection), provided that the beneficial gene can also be located on the chromosome.

Bergstrom *et al*. [223] do show, however, that plasmids can persist in two circumstances. The first is if the plasmids have a net fitness benefit and the population is subject to regular strong selective sweeps of adaptive variants immigrating into the population: in this case, bacteria that have incorporated the beneficial gene onto the chromosome are eliminated by the selective sweep, while the plasmid can transfer to the new strain. The second is if there are multiple species or ecotypes of bacteria present and the beneficial gene is occasionally lost in each ecotype (due to drift, demographic stochasticity, selective sweeps, etc.): the plasmid is maintained by the ability to transfer between ecotypes.

There has been extensive modelling of other possible resolutions of the plasmid paradox (see e.g. [221–224, 277–279]). Possible answers include selective pressures encouraging maintenance of some genes on disposable or transmissible replicons [63, 66–68, 280], nonconstant or variable rates of horizontal transfer [112, 281], the maintenance of nonconjugative plasmids by constant replenishment by conjugative plasmids that lose transfer genes [32], fitness effects of plasmid–plasmid interactions [206] and, particularly in the case of large nontransmissible plasmids close to being accessory chromosomes, evolutionary constraints of genome organization [12, 282] – after all, no one calls the presence of more than one chromosome in an organism the ‘chromosome paradox’. Future modelling work will hopefully help to tie the theories to the circumstances of specific plasmids and bacteria and determine which mechanisms are responsible for maintenance in particular systems.

Despite the wonders of plasmid biology, some bacteria are just not interested in hosting a plasmid. They instead prevent the establishment of plasmids in general or of particular plasmids using defence systems that destroy plasmids (and other mobile genetic elements) upon arrival in the cell (e.g. [161–164]). The best known bacterial immune systems are CRISPR/Cas*, which targets specific mobile genetic elements based on recorded DNA sequences, and restriction enzymes*, which target foreign DNA in general [161, 162].

### Interactions with the host community

The individual host of a plasmid will be part of a larger population of host or potential host bacteria, which in turn make up part of a larger ecological community. Since plasmids are associated with their hosts, they are inherently influenced by all community interactions. Through their effects on host phenotypes they in turn alter these interactions, and may thus be subject to eco-evolutionary feedbacks. Morever, social traits are frequently encoded by payload genes on plasmids: for example, plasmids have a higher density of genes for secreted proteins than chromosomes [165], which includes proteins involved in both cooperative and antagonistic interactions between bacteria [68]. Cooperative interactions between plasmid hosts and other bacteria include antibiotic resistance, if the mechanism of resistance is the degradation of the antibiotic [166], while antagonistic interactions include production of bacteriocins, excreted toxins that kill other bacteria and are often found on plasmids (indeed, one of the earliest discovered families of plasmids codes for colicins, bacteriocins produced by and targeting *Escherichia coli* [3]). The plasmid codes for both the bacteriocin, which is excreted (in some cases killing the host in the process), and an immunity gene, which protects against the bacteriocin [167]; thus bacteriocins behave like an extracellular version of a postsegregational killing system, killing those cells that do not carry the plasmid. Plasmid-borne genes can also play a role in interactions with other cells without actively affecting them: for example, some plasmids of *Pseudomonas* spp. sensitize their hosts to growth-inhibiting compounds excreted by other bacteria [168].

Bacterial interactions are strongly dependent on whether bacteria are free-living or in a biofilm*. Planktonic* bacteria in liquid culture can potentially encounter many different cells, but are usually not in close proximity to each other. In biofilms, bacteria are nonmotile and in close contact with their direct neighbours. Biofilms are moreover highly spatially heterogeneous, with different conditions on the surface and the interior of the biofilm, affecting the physiology and growth of bacteria. Conjugative plasmids can increase the propensity of their hosts to form biofilms (this may in part be due to the pilus serving to attach planktonic cells to the growing biofilm) [169–173]. How the aggregation of bacteria in biofilms in turn affects conjugative plasmid spread is complicated. Conjugation rates are often higher in biofilms than in planktonic bacteria due to stable cell-to-cell contact and stable mating pair formation [172], yet the spread of plasmids through biofilms is nonetheless often limited, as discussed by Stalder and Top [174]. While plasmids may not spread through the entire biofilm, the biofilm lifestyle may aid the maintenance of conjugative plasmids and prevent the loss of transfer genes [175].

Plasmids not only influence ecological interactions, but also cause genetic interactions between individuals of the host population by horizontally transferring chromosomal genes: conjugative plasmids that integrate into the host chromosome or that pick up pieces of the chromosome by homologous recombination can then transfer chromosomal DNA to a recipient on conjugation [176], such as in the classic Hfr strains of *E. coli* [3]. There is also the possibility of retrotransfer of chromsomal genes [107].

Since horizontal transfer of plasmids between distinct bacterial species is common, multispecies communities enable new plasmid dynamics, including the maintenance of plasmids in less-than-ideal hosts by transfer from a species to which they are better adapted [177], and improved compensatory amelioration of fitness costs through transfer between multiple species [142, 178]. Microbial communities also include species that are not bacteria, and plasmids can also contribute to or be affected by interactions of their hosts with these organisms. For example, Cairns *et al*. [179] showed that exposure to a virus that targets conjugation machinery to infect a cell selected for reduced conjugative ability in a plasmid, but predation by a ciliate increased conjugation rate.

### Interactions with other plasmids and mobile genetic elements

Multiple plasmids often coexist in the same population, and even a single bacterium often carries multiple plasmids of distinct types (see e.g. [37, 38, 49, 180, 181]). There is thus much opportunity for interactions between plasmids. The simplest such interaction is preventing coinfection of the same cell by excluding other plasmids. We have already seen mechanisms that can prevent coinfection: plasmid incompatibility and entry and surface exclusion [182]. Postsegregational killing can also lead to loss of a coinfecting plasmid over time [120]. Some plasmids also actively destroy other potentially coinfecting plasmids with plasmid-borne CRISPR/Cas [183–185] or restriction–modification systems [118, 119, 186] (see Fig. 2c), which work analogously to their chromosomal counterparts.

If multiple plasmids successfully establish in a common host, they may impose a high fitness cost. However, compensatory evolution that eliminates the fitness costs of one plasmid can sometimes allow for the acquisition of other related plasmids at no additional cost [187]. Similarly, some plasmids exhibit positive fitness epistasis*, meaning the total fitness cost of carrying multiple plasmids is less than the sum of the fitness costs of each individually [130, 188, 189]. Environmental conditions can affect the maintenance of coinfection: if coinfecting plasmids carry similar beneficial payload genes, the less advantageous might be lost in the presence of selection, even if coexistence is possible in the absence of selection [155]. Coinfection will have important effects on compensatory evolution to reduce plasmid fitness costs, as in coinfected hosts there will have to be evolution not only to reduce plasmid–chromosome conflicts, but also plasmid–plasmid conflicts.

The effects of plasmid interactions on horizontal transfer can be varied [190]. We have already seen that mobilizable plasmids rely on a coinfecting conjugative plasmid to be transferred; cooperative horizontal transfer can also occur through a process called conduction, in which a plasmid forms a cointegrate with a conjugative plasmid and is transferred by conjugation [114, 115, 191]; this is shown in Fig. 3(a). Between conjugative plasmids, coinfection can produce both an increase in conjugation rate (called facilitation [22]), and a decrease [192]. Similar interactions between plasmids can alter their ability to promote biofilm formation [173]. So far, we have implicitly talked about plasmid–plasmid interactions from the perspective of pairs of plasmids. When there are more than two types of plasmids present in a host cell, there will be higher-order interactions between plasmids that will need to be taken into account [156, 193].

Plasmids also coexist in their hosts with other mobile genetic elements. Some of these exist in the host in the form of a plasmid, and therefore may exhibit interactions with plasmids similar to plasmid–plasmid interactions: for example, some bacteriophages [3, 35], and integrative conjugative elements* (ICEs) [194]. Many phages target plasmid-encoded structures to infect their hosts (particularly structures related to conjugation) [195, 196]; other plasmid–phage interactions include the horizontal transmission of plasmids by transduction* in phages [22], and the ability of some plasmids to inhibit the infection of their hosts by phages [197].

## Final remarks

In this review, we provide an overview of the biology of plasmids, aiming to make the topic accessible to modellers unfamiliar with plasmids. Mathematical modelling provides a powerful tool to understand the biology of plasmids and to make predictions about their contributions to bacterial evolution (which is especially important with respect to the evolution of clinically important traits such as antibiotic resistance). The great diversity of plasmids means that modelling is needed both to seek out general unifying patterns and to determine properties specific to particular plasmid systems. We conclude by putting forward a few areas of plasmid biology that have so far scarcely been addressed by modellers.

The vast majority of plasmid models consider plasmids of only one type and distinguish only between plasmid-carrying and plasmid-free cells. Models that account for the plasmid copy number, despite appearing early on [129, 198–202], have remained overall rare. Recently the topic started to receive growing attention, mostly (but not exclusively) in the context of allele dynamics on multicopy plasmids (see Box 3 and [92]). Several of the early models of multicopy plasmids consider segregation of two incompatible plasmids and thus include coinfection of cells by multiple plasmid types. Generally, however, there are still few models that consider the coexistence of plasmids of different types in the same cell (for two examples, see [203, 204]). For a guide to designing such models, see the recent article by Igler *et al*. [197]. Studies allowing for multiple plasmid types in the population (but not within the same cell) are more common, but also rare compared to those with a single plasmid type; they mostly focus on competition between different plasmids for host cells [69, 205–208]. Coinfection and interactions between different plasmids, such as cotransfer of nonconjugative with conjugative plasmids, are common and likely have a great influence on the evolution and ecology of bacteria. To understand the evolutionary dynamics of plasmids and hosts, it is thus crucial to develop more theory that takes multiple plasmid types and coinfection into account.

A related question is why we see such great diversity of plasmids. The huge number of bacterial species and environments they inhabit implies a huge number of niches for plasmids. Yet it remains puzzling why some plasmids have a high and others a low copy number, why some plasmids are very large and others tiny, why some plasmids are conjugative and others not even transmissible, etc. Moreover, plasmids of different types coexisting in a bacterial population share the same niche, at least to a first approximation. Modelling has shown that two types of plasmids can coexist if they follow different strategies regarding their transfer rate and costs [206, 209, 210]. Similarly, the evolution of plasmid host range [147, 153, 211] and rapid speciation of plasmids [88] may contribute to the development and spread of new plasmid types in the wild. Future models could combine ecological considerations at the level of plasmids (such as differential strategies or interactions between coinfecting plasmids) with evolutionary dynamics (such as the evolution of conjugation rates or plasmid speciation) to provide further insights into the processes that shape plasmid diversity.

As pointed out in Box 2, plasmid-mediated antibiotic resistance is less studied in models than chromosomal resistance. Many models of antibiotic resistance evolution consider the effects of treatments such as combination therapies or drug dosing regimes on the evolution of resistance within hosts and strategies to manage existing resistance at the population level (for a few examples of models including plasmids, see [212–215]). Given the clinical relevance of plasmid-mediated resistance, it seems of great importance to develop further models to test how treatment regimes and population-wide protocols affect resistance on plasmids. Many infections are polymicrobial, in which case plasmids might spread from one species to others during treatment [216], influencing the treatment outcome. The effects of treatment protocols on bacterial communities harbouring plasmids are an important future direction of modelling. Resistance plasmids in commensal bacteria are also important since they may be transferred to pathogenic bacteria or the commensals themselves may become opportunistic pathogens. During treatment, commensal bacteria are often exposed to antibiotic pressure, selecting for resistance (so-called bystander selection). Moreover, plasmids carried by commensal bacteria may evolve both during treatment and in the absence of antibiotics and become less costly, for example. Transmission of commensal bacteria between individuals can spread resistance plasmids in a host population. Plasmids in commensals have already received some attention from modellers but there is much more to explore and understand (see Box 2 and also [217, 218]). Topics of concern where we see a need for further modelling include the evolution of plasmids within commensals, their maintenance in the absence of treatment, their potential transfer to pathogens, and their maintenance and spread at the population level. Of special interest are how these processes interact and how they are influenced by treatment protocols and frequencies.

Lastly, more models of bacterial biofilms are needed, both in the context of general plasmid biology and in a clinical context, taking into account a three-dimensional spatial structure with varying nutrient and antibiotic concentrations and potentially the attachment and detachment of planktonic bacteria (for an example of a biofilm model, see [219]). Plasmids are very widespread, diverse and of great relevance for bacterial ecology and evolution (and as a consequence for human health). While a large body of theory on the evolution of plasmids and their hosts exists, there remain many puzzles and unsolved questions. We hope that our review provides interested mathematical biologists with the necessary background to start to develop novel theory on plasmid dynamics.

## Glossary

**Accessory chromosome:** see secondary chromosome.

**Bacteriophage:** virus that infects bacteria.

**Biofilm:** an aggregation of bacteria that adhere to each other and a surface, and excrete a extracellular polymer that surrounds them.

**bp (base pairs**): the unit of length of a DNA molecule; a base pair consists of a pair of nucleotides from each strand of the molecule.

**Chromid:** see secondary chromosome.

***cis*-acting:** of a gene or regulatory sequence: only affecting the same replicon on which it is located; the opposite of *trans*-acting.

**Constitutively expressed:** of a gene: constantly transcribed and expressed (as opposed to being regulated so it is only transcribed in specific circumstances where it is needed).

**CRISPR/Cas:** a bacterial adaptive immune system: it consists of specific sequences from mobile genetic elements copied into the bacterial chromosome (CRISPR) and a Cas enzyme that recognizes and cuts DNA molecules with those sequences.

**Epistasis:** the interaction of different genes contributing to the same phenotype: when the collective effect of two (or more) genes on the phenotype is not just the sum of the effects to the two genes alone, there is epistasis between the two loci.

**Gene cassette:** a small mobile genetic element consisting of a gene and a site where recombination into a replicon can occur; see integron.

**Gram-positive/negative:** a major classification of bacteria, based on whether they are stained by the dye in a Gram stain procedure (named after the Danish bacteriologist Hans Christian Gram); Gram-negative bacteria have a second outer membrane surrounding their cell wall.

**Horizontal transmission/horizontal (gene) transfer:** the transfer of genetic material between two cells, as opposed to vertical inheritance of genes from mother to daughter cell.

**Integrative conjugative element:** a type of mobile genetic element: they normally exist integrated into the host chromosome, but can excise from the chromosome, take on a temporary plasmid-like form, and transfer to another cell by conjugation.

**Integron:** the place where gene cassettes are integrated into a replicon: consists of a gene coding for a site-specific recombinase and a recombination site where that recombinase can integrate gene cassettes.

**Ligation:** the process of joining the backbone of a strand of DNA together, sealing a break that previously existed between nucleotides; the opposite of nicking.

**Mobile genetic element:** any genetic material that can be transferred between locations in the genome of an organism or between organisms.

**Nicking:** the process of disconnecting the backbone of a strand of DNA by breaking the bonds between adjacent nucleotides; the opposite of ligation.

**Open reading frame:** the sequence of DNA coding for a protein.

**Phage:** see bacteriophage.

**Planktonic:** floating freely in the water column, as opposed to being associated with a surface or able to swim against the current.

**Recombination:** the process of ‘crossing over’ between two DNA molecules: the molecules are broken at the target site and the new ends are exchanged between molecules and resealed; when this occurs between two circular DNA molecules, it fuses them into one circle, and when it occurs between two parts of the same circular DNA molecule, it separates it into two circular molecules; it has site-specific and homologous forms.

**Recombination, homologous:** recombination between two double-stranded DNA molecules occurring at any location where the two molecules have a homology (that is, where their sequence is the same); most bacteria have recombinases that catalyse this process.

**Recombination, site-specific:** recombination between two DNA molecules catalysed by an enzyme that recognizes a specific sequence on the molecules to be recombined.

**Relaxase:** an enzyme that catalyses nicking.

**Replicon:** an independently replicating DNA molecule; strictly speaking, everything that replicates from a particular origin of replication.

**Repressor:** a protein that prevents the transcription (and therefore expression) of a gene.

**Restriction enzyme:** an enzyme that catalyses the breaking of a DNA molecule at a specific sequence.

**Secondary chromosome:** a large replicon in some bacteria that contains essential genes, but is smaller than the main chromosome; something between an ordinary chromosome and an ordinary plasmid.

**Segregation:** the division of copies of a replicon between daughter cells on cell division.

**SOS response:** a bacterial stress response.

***trans*-acting:** of a gene or regulatory sequence: affecting both the replicon it is located on and any homologous genes or regions on other replicons; the opposite of *cis*-acting.

**Transconjugant:** a cell that has received a plasmid through conjugation from another cell.

**Transduction:** a mechanism of horizontal gene transfer in which nonviral genetic material is accidentally packaged into a bacteriophage, and transferred by the phage to other bacteria.

**Transformation:** a mechanism of horizontal gene transfer in which free DNA molecules in the environment are taken up by a bacterium.

**Transposon/transposable element:** a mobile genetic element capable of moving itself from one location to another in the genome, either by copying or by excision and reinsertion.
